# Reproductive Management of Peruvian Grunt *Anisotremus scapularis* in Captivity: Spawning Dynamics, Hatching Rate, and Larval Survival

**DOI:** 10.3390/ani15111579

**Published:** 2025-05-28

**Authors:** Jordan I. Huanacuni, Renzo Pepe-Victoriano, Pablo Presa, Luis A. Espinoza-Ramos

**Affiliations:** 1 Área de Biología Marina y Acuicultura, Facultad de Recursos Naturales Renovables, Universidad Arturo Prat, Arica 1000000, Chile; rpepev@unap.cl; 2 Programa de Magister en Acuicultura: Mención en Cultivos de Recursos Hidrobiológicos y Mención en Acuiponía, Facultad de Recursos Naturales Renovables, Universidad Arturo Prat, Arica 1000000, Chile; 3 Finfish Aquaculture—Sociedad Anónima Cerrada, Tacna 23004, Peru; 4 Núcleo de Investigación Aplicada e Innovación en Ciencias Biológicas, Facultad de Recursos Naturales Renovables, Área de Biología Marina y Acuicultura, Universidad Arturo Prat, Iquique 1110939, Chile; 5 Laboratory of Marine Genetic Resources (ReXenMar), CIM—Universidade de Vigo, 36310 Vigo, Spain; pressa@uvigo.gal; 6 Escuela de Ingeniería Pesquera, Universidad Nacional Jorge Basadre Grohmann, Tacna 23000, Peru; lespinozar@unjbg.edu.pe

**Keywords:** broodstock, candidate species, marine aquaculture, reproductive rates, spawning dynamics

## Abstract

The Peruvian grunt, *Anisotremus scapularis*, is a commercially valuable coastal fish in the southeastern Pacific that is facing overexploitation. To support its aquaculture development, this study evaluated the spontaneous reproductive dynamics of a captive broodstock held under natural photoperiod and temperature conditions in a flow-through system. Eleven wild adult specimens (3 females and 8 males) with an average size of 34.9 ± 5.4 cm and a weight of 986 ± 470 g were housed in a 9 m^3^ tank and monitored over five consecutive spawning seasons (2016–2021). Fish were fed a semi-moist, animal-protein-based diet (37% protein and 6.6% lipid) at 2% body weight/day. A total of 214 spontaneous spawning events produced over 83 million eggs. The highest reproductive output occurred in the first season (2016–2017) with 94 spawnings and 23.3 million eggs. Fertilization, hatching, and larval survival rates averaged 94.7%, 89.7%, and 75%, respectively, but declined in later years. Spawning showed marked seasonality from October to May, with a major reproductive pause in late January. The temperature (16–20 °C) and photoperiod (>12 h daylight) appeared to influence reproductive timing, alongside diet and broodstock handling. The findings reported herein are observational in nature and provide valuable baseline data for future experimental designs aimed at optimizing broodstock management in *A. scapularis* aquaculture.

## Simple Summary

The aquaculture of new marine species faces significant challenges in managing and optimizing the reproductive cycle in captivity. Broodstock control is crucial for success, especially in emerging aquaculture species such as the Peruvian grunt (*Anisotremus scapularis*), a coastal fish of great importance in Peruvian artisanal fisheries. This research covered five consecutive spawning seasons (2016–2021) of a grunt broodstock whose natural spawning began in the seventh month post-capture and exhibited marked annual reproductive seasonality between early spring (October) and mid-autumn (May). The spawning pattern was characterized by a significant reproductive pause in late January and shorter spawning interruption intervals every 30 days. The most abundant laying (23,280,055 eggs) was observed in the 94 spawning events of the first reproductive season in captivity (2016–2017). The hatching rate and survival rate reached 94.67% and 75%, respectively. All reproductive efficiency rates were significantly better in early annual seasons than in later annual seasons as the broodstock aged. We show that the reproductive cycle of *A. scapularis* is fundamentally influenced by manipulative interventions, nutrition, and the temperature. Controlling these factors allows for a predictable and consistent supply of offspring, which in turn facilitates the planning and management of aquaculture operations off the coasts of southern Peru and northern Chile.

## 1. Introduction

The success of marine aquaculture depends largely on the ability to reproduce fish species reliably and efficiently in captivity. Reproductive control in broodstock is a cornerstone of hatchery production, as it enables the planning of spawning events, larval availability, and overall production scheduling [1,2]. Controlled reproduction also reduces reliance on wild seeds, enhancing sustainability and traceability in aquaculture operations [3,4].

The Peruvian grunt (*Anisotremus scapularis*) is a carnivorous coastal fish native to the southeastern Pacific Ocean, ranging from Ecuador to northern Chile [5]. It inhabits intertidal and subtidal rocky reefs and forms large schools in shallow coastal waters [6]. This species holds significant cultural and economic value, as it has been consumed since pre-Hispanic times [7,8] and currently forms a part of artisanal fisheries in Peru and Chile [9,10]. In recent years, increasing fishing pressure, the lack of effective management, and habitat degradation have led to signs of overexploitation in *A. scapularis* populations, as reported by fishery assessments [11,12]. While fishery regulations such as catch limits and gear restrictions are important, aquaculture emerges as a complementary strategy to alleviate fishing pressure and support stock enhancement [13]. However, the aquaculture of *A. scapularis* is still in its nascent stage and faces critical bottlenecks, particularly in the reproductive phase.

Advances have been made in larval rearing [14], feed enrichment [15], and juvenile growth under different stocking densities [16]. Yet, broodstock management, specifically long-term spawning dynamics under captivity, remains poorly documented. Available information suggests that reproductive seasonality in *A. scapularis* is influenced by temperature and photoperiod [17,18], but these conclusions are often based on short-term trials or wild fish samples. Maintaining *A. scapularis* broodstock under captivity for several consecutive reproductive seasons presents biological and operational challenges. Unlike well-domesticated species such as seabream (*Sparus aurata*) [19], the common carp (*Cyprinus carpio*) [20], or tilapia (*Oreochromis niloticus*) [21], the reproductive physiology of *A. scapularis* in controlled environments remains underexplored [18]. Knowledge gaps persist regarding its reproductive longevity, the timing of spawning under natural conditions, and the performance of gametes and larvae throughout multiple seasons.

Additionally, the existing literature sometimes implies levels of reproductive control (e.g., hormonal induction or environmental manipulation) that have not yet been established for this species. Thus, there is a need for baseline observational data that describe what occurs when wild-caught broodstock is held in semi-controlled, flow-through systems under natural seasonal variation. The present study addresses this gap by describing the spontaneous spawning dynamics of *A. scapularis* broodstock over five consecutive reproductive seasons (2016–2021). Wild-caught adults were maintained in a single 9 m^3^ flow-through tank without environmental manipulation or hormonal induction. We recorded the spawning frequency, total egg production, fertilization and hatching rates, and early larval survival. We also examined how these parameters varied over time and under changing natural conditions of temperature and photoperiod. The aim was to establish reference reproductive patterns that can inform future work on reproductive control and hatchery planning for this emerging aquaculture species.

## 2. Materials and Methods

### 2.1. Broodstock Maintenance

**Figure 1:**
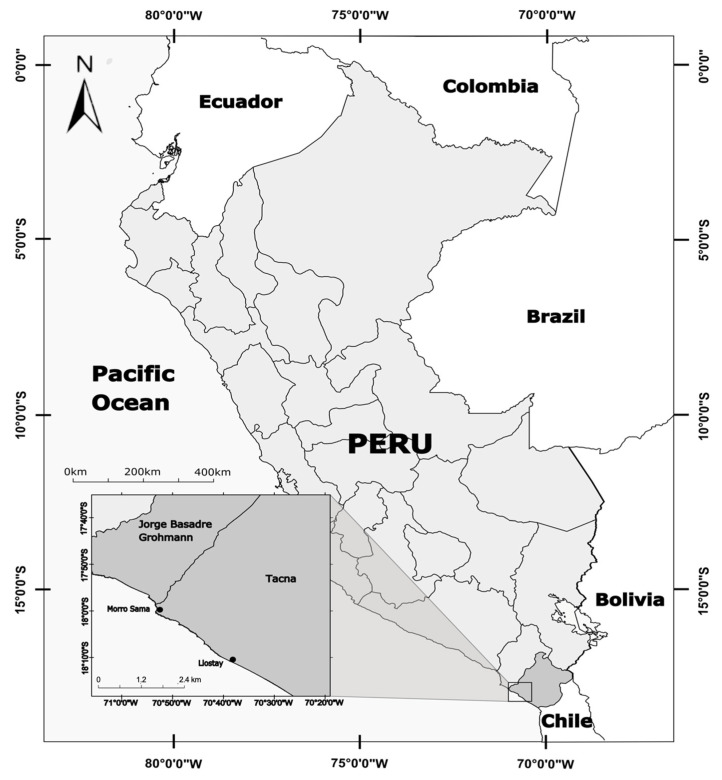
Collection site (Llostay beach) and conditioning site (Morro Sama) of *A. scapularis* adults in Tacna, Peru.

The research was carried out between March 2016 and August 2021 at the Morro Sama Aquaculture Center (CAMOSA) of the National Fund for Fisheries Development (FONDEPES) in the Tacna region, Peru (17°59′39.7′′ S, 70°52′59.1′′ W) (Figure 1). In that facility, a circular Australian-type tank with a volumetric capacity of 9 m^3^ (4 m in diameter and 1 m in height) was prepared to house wild *A. scapularis* adult breeders. The tank was made of corrugated steel plates covered with a black polyvinyl chloride (PVC) geomembrane and was conditioned with constant entry and exit of seawater to the hatchery (flow-through system) at a flow rate of 75 L/min (0.5 water renewal/hour). The seawater used to supply the broodstock tank was collected directly from the CAMOSA intertidal zone, which is characterized by its high oxygenation and stable physicochemical parameters. Before use, the collected water was decanted to remove suspended solids and organic debris.

**Figure 2:**
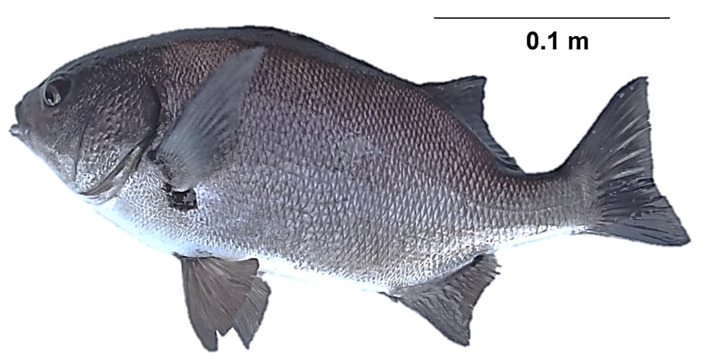
Photograph of an adult male *A. scapularis* used as a breeder in experimental reproductive trials. External sexual dimorphism was not evidenced.

Three hook-and-line fishing campaigns using *Emerita analoga* as the bait were carried out on the rocky beaches of Llostay, Tacna, Peru (18°10′26′′ S, 70°38′37′′ W), between March and April 2016, capturing 15 adult specimens of *A. scapularis* (Figure 2). The weight of the captured fish was recorded using a digital scale (Makita, 0.1 g accuracy, Makita Electric Works, Ltd., Anjo, Japan) and the total length using an ichthyometer. These fish were transported to CAMOSA in a 1 m^3^ fiberglass cubic container equipped with pure oxygen and ice, containing 500 L of raw seawater tempered at 19.5 ± 0.5 °C [22]. Fish were placed in the pre-conditioned Australian tank and fasted for 7 days after capture, receiving preventive treatments with oxytetracycline at 50 ppm and formalin at 61 ppm. Three specimens died from injuries inflicted upon their capture, and one failed to adapt to the culture condition. Sexual identification was performed in September 2016, prior to the first spawning. Eight males were identified via the release of sperm under gentle abdominal pressure, while three females were identified due to the presence of swollen abdomens. The release of transparent oocytes was used to confirm ovulation. After five months in captivity, the fish averaged 34.91 ± 5.37 cm in total length and 0.986 ± 0.470 kg in weight. In this body condition, fish are expected to reach sexual maturity, i.e., at least 1.97 years of age and 21 cm in length [12]. The experiment was carried out with 11 specimens with a sex ratio of 3:8 (female/male).

The natural photoperiod and water temperature were recorded daily between 2016 and 2021. Seasonal temperature variations in the culture system were recorded and compared to typical values in the species natural habitat. As a flow-through system, thermal changes in the tanks were observed to follow a pattern similar to that of coastal waters, with a temperature range of 23.3 °C in summer and 13 °C in winter. This fluctuation reflects environmental conditions remarkably similar to those experienced by fish in their natural environment. During the five spawning seasons, the natural photoperiod was used over the broodstock tank. The tank was covered by a Raschell mesh dome, which allowed sunlight to enter year-round through a small window, providing up to 300 lux at the water surface. Salinity and dissolved oxygen were monitored daily, with one salinity measurement per day and dissolved oxygen measurements every six hours. The water temperature was recorded every six hours to assess its daily variability, while the photoperiod was recorded daily to ensure consistent lighting conditions in the culture system. Water temperature conditions depended on seasonality, ranging from 13 °C to 22.3 °C, salinity from 35 to 37 ups, and oxygen from 4.3 to 7.9 mg/L.

The dietary adaptation of the fish was initiated with frozen crustacean *Emerita analoga* for 38 days (18 March to 25 April 2016), followed by *Emerita analoga* impregnated with fishmeal for 34 days (26 April to 24 May 2016). The final semi-moist maintenance diet (from June 2016 until the end of this study) was formulated biweekly with the following proportions: anchovy (*Engraulis ringens*) meal (50%), anchovy oil (4%), soybean meal (20%), wheat flour (25%), trace minerals and vitamin premix (0.1%), and powdered colapis (0.9%). The nutritional composition was determined weekly via proximate analysis following AOAC (Association of Official Analytical Chemists) methods, protein via the Kjeldahl method (method 2001.11), lipid via the Soxhlet method (method 2003.05), ash via incineration (method 942.05), fiber via an enzymatic–gravimetric method (method 985.29), and moisture via drying (method 925.10). The averaged composition was protein 37.1 ± 0.9%, fat 6.6 ± 0.3%, ash 9.5 ± 0.2%, fiber 0.7 ± 0.1%, and moisture 44.3 ± 1.1% [22]. The pelleted feed was stored frozen at −10 °C to preserve its freshness and prevent microbial growth. Before feeding, the required amount of feed was thawed and poured into the culture tank. Fish were fed a daily feeding rate (DFR) of 2% of their total biomass using the semi-moist diet throughout the experiment. Biometry allowed for monthly adjustments for the first six months, and annual biometry was performed thereafter to calculate the 2% DFR, as shown in Table 1. This annual biometry was performed to avoid excessive handling of the broodstock, which might cause stress and delay spawning. In addition to the semi-moist feed, 10 g of ranga-ranga (beef belly) and 15 g of piure (*Pyura chilensis*) per fish were fed daily from one month before the start of the spawning season.

### 2.2. Natural Spawning and Egg Collection

The onset of the spawning season, which occurs in the early Southern Hemisphere summer, was intuited from historical reproductive records of Southeast Pacific rockfishes such as *Helcogrammoides chilensis* [23]. To assess gonadal maturation and spawning onset, visual inspections were conducted during monthly biometry for the first six months to detect changes in the abdomen of the fish, which are indicative of advanced gonadal development. Indicators of reproductive behavior such as the interaction between individuals, swimming in schools, increased courtship activity, gamete release, and the presence of floating eggs in the broodstock tank were also useful. *A. scapularis* was reared in captivity without hormonal treatment. At the beginning of the spawning season, a cylindrical egg collector made of 300 μm planktonic mesh with a circular ring at the top, fitting into a rigid vertical frame measuring 0.3 × 0.3 × 0.6 m, was placed at the outlet of the broodstock tank overflow [1]. The egg collector was placed in the tank throughout the year from 3:00 p.m. onwards, as recurrent spawning began daily between 5:00 p.m. and 7:00 a.m. Eggs were carefully transferred into a 20 L bucket and transported to the CAMOSA hatchery. In the incubation room, eggs were washed on a 300 μm sieve with sterilized seawater, filtered to 1 μm with a cartridge filter, and treated with ultraviolet (UV) light in an AL-PVC-160W system (America Ultraviolet, Temecula, CA, USA).

### 2.3. Egg Quality, Hatching Rate, and Survival

Eggs were collected and placed in an aerated 20 L bucket for up to 2 h before being assessed for viability. Collected eggs were transferred to a 1 L test tube and allowed to stand for 15 min. Floating eggs were considered viable, while those that sank were considered non-viable [1], and their sum constituted the total number of eggs. Non-viable eggs (735 µM in diameter) settled at the bottom, forming a well-identifiable layer, and were placed in a new graduated 1 L cylinder to quantify them. The quantification factor of 738 egg/mL was determined as the average of 20 1 mL egg samples from a homogeneous suspension and counted under a stereomicroscope. Meanwhile, viable eggs (800 µM in diameter) were immersed for 5 min in 4 L of a 1.5% dilution of iodine antiseptic (AQUAYODO^®^, Veterquímica, Lima, Peru), concentrated to 2 mL/L of seawater, and placed into a 500 L black fiberglass tank with 400 L of seawater and a gentle air supply. Six 130 mL water samples were randomly collected from different points in the incubation tank to quantify the number of viable eggs per milliliter using a stereomicroscope.

Viable eggs were incubated for four days with a daily partial replacement (9:00 a.m.) of 30% seawater using a 300 μm sieve. After 48 h of incubation, the average number of hatched larvae was estimated from six 130 mL water samples taken from different points in the incubation tank. The hatching rate (HR, %) was calculated with Equation (1) using the extrapolated number of hatched larvae to the total volume of the incubation tank.

(1) $$Hatching\;rate\;(\%)=\;\;\frac{Hatched\;larvae}{Viable\;eggs}\times 100\%$$

At the end of four days of incubation (48 h post-hatching or 96 h of incubation), live larvae (larvae with yolk consumption and ocular pigmentation) were quantified to determine the survival rate (SR, %) according to Equation (2). The survival assessment was carried out at 48 h post-hatching, a time when 100% of the larvae had the yolk absorbed in previous tests. This is a relevant characteristic of haemulids, which present accelerated yolk consumption and a short larval development period [24]. The procedure consisted of six random samples of 130 mL each that were collected from the incubation tank using a beaker, their transfer to a Petri dish under a stereomicroscope to count the number of live larvae, and the final scaling of the average number of larvae counted to the total number of live larvae in the tank using Equation (2):

(2) $$Survival\;rate\;(\%)=\;\;\frac{Live\;larvae}{Hatched\;larvae}\times 100\%$$

### 2.4. Data Analyses

Statistical analyses were performed using RStudio, version 2024.09.0+375, from RStudio, Inc. (Washington, DC, USA). The normality of all data was assessed using the Anderson–Darling test, and homogeneity of variance was assessed using Bartlett’s test. Data were evaluated using one-way analysis of variance (ANOVA) and Tukey’s post hoc test. When normality assumptions were not met, data were evaluated using the Kruskal–Wallis test to assess differences between treatments and Dunnet’s test to determine group homogeneity [25]. Graphs were created using the ggplot2 package and expressed as the mean ± standard deviation (SD).

## 3. Results

### 3.1. Annual Spawning Dynamics

**Figure 3:**
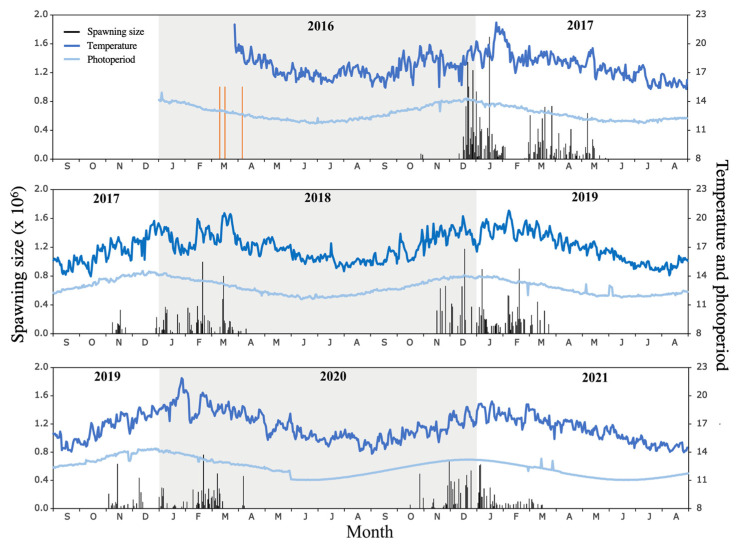
Monthly spawning size (number of eggs), temperature (°C), and photoperiod (daylight hours) during the period of 2016–2021 in the *A. scapularis* broodstock tank. Orange line represents capture of wild fish.

From October 2016, after seven months under indoor conditioning, the broodstock began its daily spawning between 5:00 p.m. and 7:00 p.m. (with the majority in that interval) in a temperature range of 18–20 °C, a natural light intensity of 335.85 lux, and a water flow rate of 0.61 L/s. The first natural spawning of the *A. scapularis* broodstock extended from 29 October 2016 to 16 May 2017 (203 days), encompassing 94 spawning events, with 25 and 20 events in January and March, respectively, and a total of 23,280,055 eggs, e.g., December (6,776,284 eggs), January (6,903,141 eggs), and March (5,208,345 eggs) (Figure 3 and Table 1).

The 2017–2018 spawning season ran from 8 October 2017 to 10 April 2018 (155 days, 54 spawnings), with a total of 9,476,836 eggs. The 2018–2019 spawning season ran from 10 October 2018 to 24 March 2019 (130 days, 56 spawnings), with a total of 15,438,822 eggs. The 2019–2020 spawning season ran from 4 November 2019 to 8 April 2020 (157 days, 57 spawnings), with a total of 8,832,161 eggs. The 2020–2021 spawning season extended from 27 October 2020 to 17 March 2021 (153 days, 58 spawning events), with a total of 10,192,337 eggs (Table 1).

The maximum number of eggs recorded in the period of 2016–2021 was on 16 January 2017 (1,693,808 eggs), with a mean monthly temperature and photoperiod of 19.73 ± 1.29 °C and 13.74 ± 0.15 daylight hours, respectively. The dynamics of spontaneous spawning of *A. scapularis* in captivity over five seasons showed a fluctuation in maximum spawning peaks, i.e., usually in February but occasionally in December–January. This spawning pattern was characterized by a long reproductive pause in late January or early February (Figure 3). Shorter intervals of spawning interruption were observed approximately every 30 days, except in years with significant November spawning (e.g., 2017), where a prolonged reproductive pause (approximately 30 days) was observed in December.

**Figure 4:**
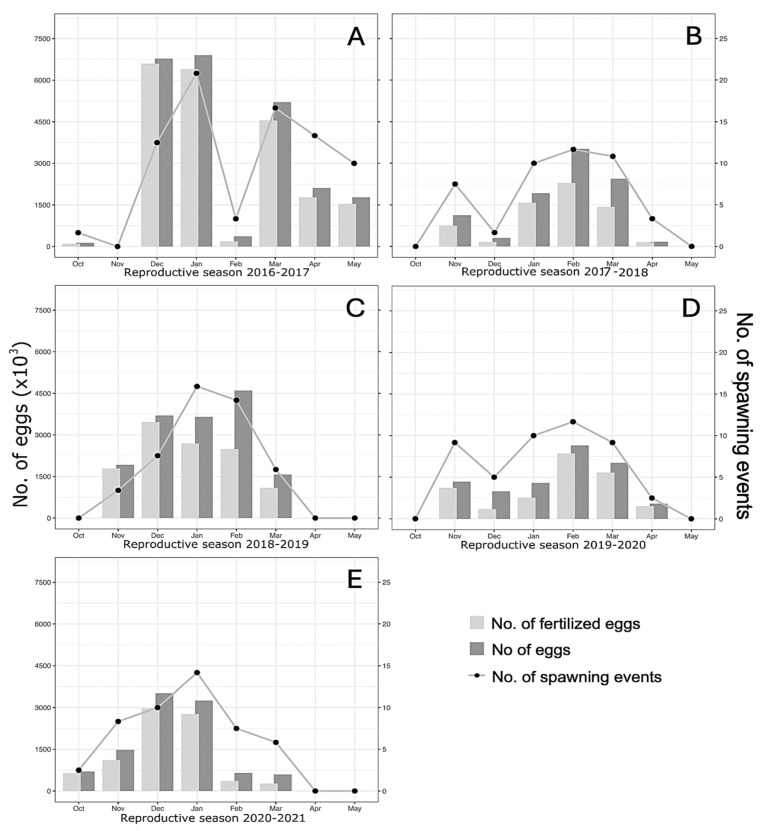
Data from five spawning seasons (**A**–**E**) of the *A. scapularis* broodstock. Each graph shows the number of spawnings per month (right axis) and the number of eggs laid (left axis). The graphed data represent the number of fertilized eggs (light gray bars), the number of eggs (dark gray bars), and the number of individual spawnings (dot and line plots). Monthly values are based on the spawning performance of the captive broodstock maintained under natural photothermal conditions.

The mean monthly spawning size across seasons ranged from 1.47 × 10^6^ (2016–2020) to 3.33 × 10^6^ (2016–2017), with no statistical differences between seasons (Table 1). The total number of eggs laid, monthly spawning events, and fertilized eggs (e.g., Anderson–Darling test: *A* = 0.992 and *p* = 0.01) did not conform to a normal distribution within the five reproductive seasons of *A. scapularis* (Figure 4). Despite apparent differences, no statistical differences were obtained between spawning seasons for the No. of eggs, the number of spawning events, and the number of fertilized eggs (Kruskal–Wallis test: 4.864 and *p* = 0.301).

### 3.2. Temporal Patterns of Spawning Efficiency

The mean number of fertilized eggs (FEs) per month did not vary between seasons or months. The hatching rate ranged from 66% to 79%, with no statistically significant differences between spawning seasons. Significant differences in survival rate were observed among the five spawning seasons, being highest in the 2016–2017 season and lowest in the 2019–2020 and 2020–2021 seasons (Table 1).

**Figure 5:**
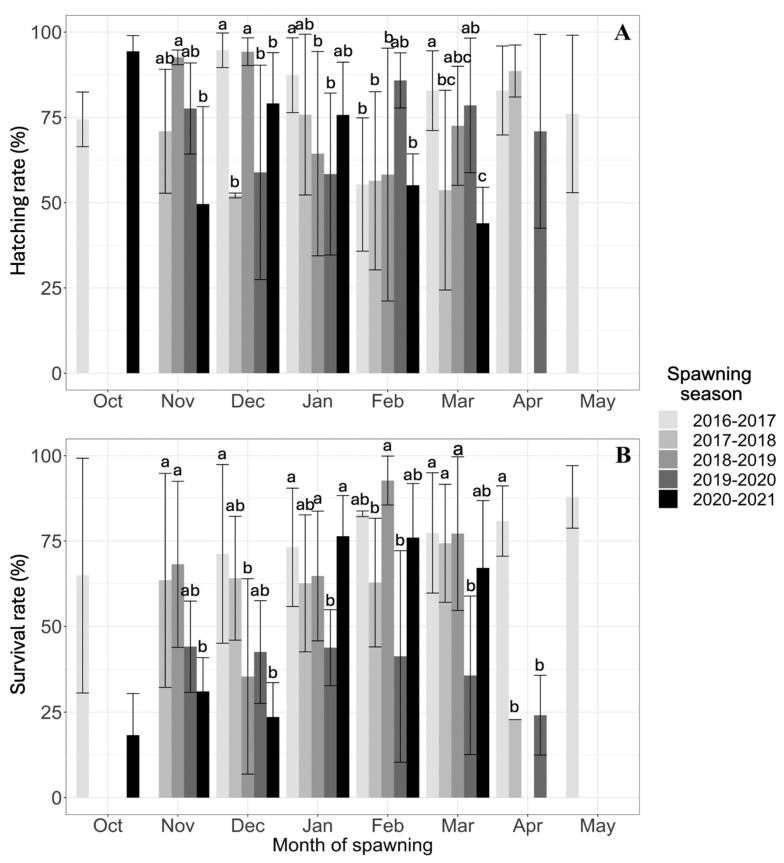
Data on five breeding seasons of *A. scapularis* broodstock are represented by the mean monthly hatching rate (HR, %) and survival rate (SR, %). The same letter (a, b, and c) on the means of the intramonthly distributions indicates no significant differences in the HR or SR between spawning seasons (2016–2021). (**A**) Hatching rate; (**B**) Survival rate.

The hatching rate (TR) of *A. scapularis* was normally distributed across all breeding seasons (Anderson–Darling test: *A* = 2.690 and *p* = 6.58–0.7). The lowest monthly TR in the historical series (43.96 ± 10.55)% was observed in the last month of the 2020–2021 season. The highest TRs were observed in October 2020 (94.37 ± 4.61)%, in December 2016 (94.67 ± 5.08)% and 2018 (94.25 ± 4.05)%, in February 2020 (85.85 ± 8.08)%, and in April 2017 (88.60 ± 7.63)%. Despite significant point differences in HR between months within the season, there was no clear trend in HR (Figure 5).

The larval survival rate (TS) of *A. scapularis* conformed to a normal distribution across all reproductive seasons (Anderson–Darling test: *A* = 1.6495 and *p* = 2.59–0.4). The lowest TS (18.30 ± 12.16)% was observed in October 2020. The highest TS was observed in February 2017 (82.97 ± 0.83)% and 2019 (92.72 ± 7.17)%, in March 2018 (74.34 ± 17.29)%, in November 2019 (44.09 ± 13.31)%, and in January 2021 (76.43 ± 11.89)% (Figure 5). Despite significant differences in larval SR between spawning seasons, no differences were observed between months within the spawning season, except for the lower SR in the spring months than in the summer months of the 2020–2021 season (K-W test = 30.27 and *p* = 0.000013).

### 3.3. Relationship Between Spawning and Abiotic Parameters

The annual temperature and photoperiod showed a normal distribution across the five laying seasons (Anderson–Darling test: *A* = 0.308 and *p* = 0.5461; *A* = 0.333 and *p* = 0.498, respectively). As expected, the mean values of the temperature and photoperiod showed significant differences between months within a season (*F* = 7.791 and *p* = 1.68–0.5; *F* = 18.94 and *p* = 1.10–0.9, respectively). The highest values of temperature and photoperiod were observed in the 2016–2017 breeding season, and the lowest values were observed in the 2017–2018 and 2020–2021 seasons (Figure 3).

**Figure 6:**
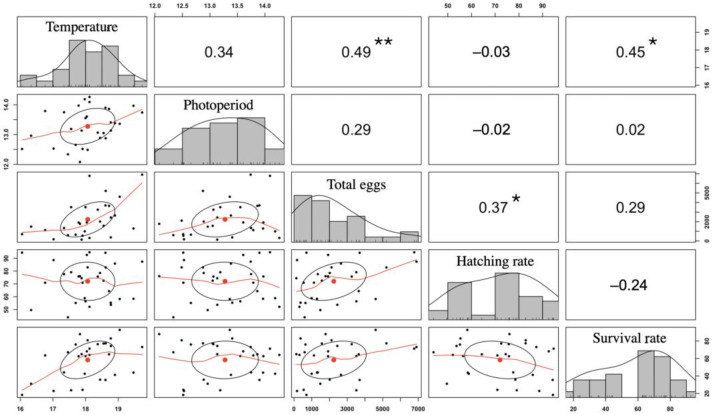
Scatter plot with distributions and multiple correlations between the environmental variables water temperature (°C) and daylight photoperiod (h) and the reproductive variables total number of eggs (%), hatching rate (%), and survival rate (%) in the *A. scapularis* broodstock tank in the period of 2016–2021. The distribution of each variable is represented on the main diagonal. Below the diagonal, bivariate relationships with their linear regression lines are shown. Above the diagonal, correlation coefficients between variables are shown. Significance thresholds are as follows: * *p* < 0.05, ** *p* < 0.01.

The total No. of eggs and the hatching rate did not conform to normality and did not differ between seasons (K-W test = 4.876 and *p* = 0.301) or between months (K-W test = 43.916 and *p* = 0.418). The survival rate (SR) did not conform to normality and differed marginally between its minimum in season 2019–2020 and the rest of the seasons. Those three parameters did not differ between months throughout the 2016–2021 period. The water temperature was positively correlated with the total number of eggs spawned (*r*^2^ = 0.49) as well as with the survival rate (*r*^2^ = 0.45) (Figure 6). The total number of eggs was positively correlated with the hatching rate (*r*^2^ = 0.37) (Figure 6; Supplementary data).

## 4. Discussion

This study describes the reproductive dynamics of *A. scapularis* broodstock during five spawning seasons under natural temperature and photoperiod conditions. Knowledge of these dynamics is essential for planning the reproductive logistics in the hatchery of this candidate species for aquaculture in the South Pacific region. We describe the manipulation conditions necessary to trigger annual spawning cycles in controlled systems, which constitutes a fundamental advance to achieve synchronized spawning events, the continuous collection of spawning supplies, and the maximization of egg and sperm efficiency, as has been achieved in other species, such as the tiger grouper (*Epinephelus fuscoguttatus*) [26]. It should be noted that this study is observational in nature, based on long-term monitoring of a single broodstock maintained in a tank under natural photothermal conditions. Therefore, the descriptive findings provide fundamental information on natural spawning dynamics, which can inform future experimental designs and modulate spawning timing.

One of the keys to success has been the minimal manipulation of the fish to avoid interfering with their complex behavioral changes, both in the early stages of domestication and during the reproductive season [27,28]. This conclusion is deduced from the fact that the broodstock had natural and stable spontaneous spawnings for five consecutive seasons, without resorting to stressful spawning or spawning induction [29]. The other key to reproductive success was the progressive diet used from the capture of adults in March 2016 to the semi-moist diet of the broodstock from June 2016, resulting in a balanced and nutritious diet to meet the energetic demands of gamete development and spawning [3]. This conclusion is deduced from the general health of the broodstock over five generations, which makes them more resistant to stress and disease, as well as from the high hatching rates and larval survival observed thereafter.

### 4.1. Annual Pattern of Spawning Dynamics

The spontaneous spawning of *A. scapularis* began when an increase in temperature and daylight hours became evident in the South Pacific region. This is logical, given that most farmed species are poikilothermic and temperature variation significantly influences their reproductive physiology [30,31,32]. The spring-like environmental conditions of the South Pacific region determine the marked reproductive seasonality of this species, as also observed in other species in the wild, as well as in those bred in captivity [33,34]. Since this is a spontaneous spawning event, it is highly likely that the spawning period in the culture of this species does not differ from the natural one, as observed in other species, such as the reddish-orange medaka (*Oryzias latipes*) [35].

Aquaculture under controlled conditions requires knowledge of the triggering photoperiod. During the five reproductive seasons studied, spawning was observed in a photoperiod range of 12/12 to 14/10 day/night, and no spawning was observed in the 11/13 day/night regime. Notably, the absence of spawning has also been observed in the 11/13 day/night photoperiod at higher Peruvian latitudes, with the stimulation of vitellogenesis and spawning starting from the 13/11 day/night regime [17]. This regional variation has also been demonstrated in the duration of the embryonic development phases of this species [18]. A similar phenomenon has been reported in species such as the small yellow croaker (*Larimichthys polyactis*) [36] and is crucial for the adaptation of regional aquaculture conditions.

The number of eggs laid depends on the spawning season [37], but spawning patterns in the Peruvian coastal ecosystem can exhibit large variability in latitude, between years, and interspecifically. For example, the Peruvian anchovy (*Engraulis ringens*) spawns year-round, but with a main peak between August and October and a secondary peak between February and March. The spontaneous spawning dynamics of *A. scapularis* in captivity during five seasons in southern Peru showed its maximum spawning peaks either in December–January and/or in February–March (e.g., 2016–2017). However, the large annual standard deviation of the number of eggs, the number of spawning events, and the number of fertilized eggs did not allow for statistical differences between years. That is, *A. scapularis* began its natural spawning in early spring (October) and continued until mid-autumn (May), encompassing the spawning period reported in central Peru [38]. Within that seasonal spawning range, the highest reproductive activity lasted five months, usually from November to March, with maximum larval performance and survival in February. The species performed a spawning pause approximately every 30 days, with a major reproductive pause in late January or early February. However, in years with significant early spawning, for example, November 2017, the major reproductive pause (approximately 30 days) was observed in December. Knowledge of these intervals is an asset for aquaculture management, influencing production and reproduction strategies, as it is being customized in the aquaculture of several species such as sturgeons [39] or in the three-spined stickleback (*Gasterosteus aculeatus*) [40].

### 4.2. Temporal Patterns of Spawning Efficiency

Despite the low fertilization rates (FRs) commonly reported in captive marine fish [41], in *A. scapularis*, we have observed high FRs ranging between 44% and 94%. Furthermore, the high hatching rates (HRs) observed (66% and 79%) did not show a temporal trend, and their maxima were distributed between October and April, depending on the spawning volume of each season. Provided that the egg number was positively correlated with the HR and this was significantly influenced by temperature, we believe that temperature is the main determinant of spawning efficiency.

Current high hatching rates were observed under the same incubation temperature as that used in spontaneous spawning. It is known that when eggs are incubated at a temperature above the optimum, the yolk sac is absorbed rapidly, causing premature hatching of the larvae [42], but when eggs are incubated at temperatures below the optimum, there is a delay in embryo development and an increase in susceptibility to diseases and reduced viability [43]. For example, this has been observed in the common carp (*Cyprinus carpio*) which shows a negative correlation between the hatching rate and elevated temperatures [44].

Similar to the hatching rate (HR), the maximum survival rate (SR) was observed in different months, depending on the spawning season and water temperature. This direct relationship between the SR and temperature was observed both within seasons (e.g., 2020–2021, where the SR was lower in spring months (16.64 ± 0.80) °C than in summer (17.84 ± 0.47) °C) and between years (e.g., a higher SR in the first spawning season (2017–2018) (18.42 ± 0.63) °C and lower in the last one (2020–2021) (17.08 ± 0.83) °C). Notably, a higher SR with temperature also increased subsequent larval and juvenile growth in other species, e.g., the Atlantic cod (*Gadus morhua*) [45]. The observed variability in larval survival rates between years could be attributed to fluctuations in temperature and dissolved oxygen levels during incubation and early larval stages [46]. Furthermore, the bloodstock’s age and reproductive senescence can affect gamete quality and, consequently, larval fitness [47]. Also, the nutritional status of the broodstock in February, when peak spawning occurred, could have contributed to variation in larval survival rates thereafter. These hypotheses merit further investigation on the reproductive biology of *A. scapularis*.

### 4.3. Relationship Between Spawning and Abiotic Parameters

The positive correlation between the water temperature, the total number of eggs laid, and the survival rate in *A. scapularis* suggests that temperature is an environmental trigger for spawning; that is, it is crucial in gametogenic processes. For example, this has been demonstrated by measuring yolk accumulation in teleost eggs [48], as well as larval survival and production in high-quality fish, such as the yellow flounder (*Pleuronectes ferrugineus*) [49]. Interestingly, a positive relationship between egg number and larval survival has been reported in other groupers [50]. However, in the present work, no such correlation was observed, but a positive correlation was observed between the total egg number and hatching rate. It is known that a high egg number increases the probability of successful hatching, and hatching is modulated by factors such as female quality, including age, physiological condition, and reproductive history [51]; therefore, we believe that the optimal abiotic and nutritional environment of *A. scapularis* spawners likely influenced the observed correlation between the total egg number and hatching rate, while survival would depend on many other external factors. It is also well known that the photoperiod can stimulate egg and sperm maturation, preparing spawners for spawning [52]. Because *A. scapularis* depends on seasonal changes to time its reproduction, we expected a correlation between the temperature and the photoperiod in all five reproductive seasons involved in this study. However, such a relationship was not observed. This suggests that modulating the temperature may be more effective than controlling the photoperiod for spawning year-round outside the natural breeding season, as is performed in other aquaculture species such as tilapia (*Oreochromis niloticus*) [2].

## 5. Conclusions

Our study provides valuable information on the reproductive control of *A. scapularis* broodstock as a prelude to its industrial aquaculture. This control allows for a predictable and constant supply of offsprings, which in turn facilitates the planning and management of aquaculture operations. Specifically, we present key information on the nutrition, temperature, and photoperiod to trigger and stabilize the annual spawning of *A. scapularis*. We report that the main spawning activity lasts five months, generally from November to March, and minor spawning intervals are predictable every 30 days, while a longer interval depends on the temperature at the beginning of the spawning season. A temperature in the range of (16–20) °C and a photoperiod above 12/12 allowed indoors spawning when these conditions were accompanied by a diet based on animal protein sources and a semi-moist feed formulated with 37.13% protein content. There are two other key aspects to the reproductive strategy of this species that also need to be considered. One is the necessary research on the application of the thermophotoperiod in a controlled system to ensure a continuous supply of eggs throughout the year. This is key because events such as El Niño or sharp increases in sea temperature cause spawning and productivity to be advanced or delayed in aquaculture systems [53]. The second aspect to be controlled is the genetic diversity of broodstock, since its maintenance is essential to prevent inbreeding depression, which can lead to reduced fertility and increased susceptibility to disease [54]. Finally, the incipient aquaculture of this species may help alleviate the overexploitation of the *A. scapularis* population, which should be carefully evaluated, as it represents the original genetic source of captive broodstock. The authors urge relevant authorities and institutions to collaborate to promote sustainable fisheries in Peru and, at the same time, develop subsidiary aquaculture.

## Figures and Tables

**Table 1:** Characteristics of five spawning seasons of the *A. scapularis* broodstock from 2016 to 2021. S, number of spawning events; D, length of the spawning season (days); L, mean adult length; W, mean adult weight; No. eggs, total number of eggs per season; SS, mean spawning size per month; FE, mean number of fertilized eggs per month; HR, mean hatching rate; SR, mean survival rate; and SD, standard deviation. The same letter (a, b, and c) on the mean figures implies no significant differences between spawning seasons.

Spawning Season	S	D	L ± SD (cm)	W ± SD (g)	No. Egg	SS ± SD (×10^6^ Eggs)	FE ± SD (×10^6^ Eggs)	HR ± SD (%)	SR ± SD (%)
2016−2017	94	203	37.00 ± 5.21	1236.50 ± 745.73	23,280,055	3.33 ± 2.92 ^a^	3.02 ± 2.79 ^a^	79.07 ± 12.49 ^a^	75.09 ± 7.78 ^a^
2017−2018	54	155	37.50 ± 5.08	1310.25 ±788.36	9,476,836	1.58 ± 1.30 ^a^	1.06 ± 0.85 ^a^	66.25 ± 14.62 ^a^	58.38 ± 17.98 ^ab^
2018−2019 ^1^	56	130	-	-	15,438,822	3.09 ± 1.29 ^a^	2.30 ± 0.90 ^a^	76.39 ± 16.37 ^a^	67.65 ± 21.02 ^abc^
2019−2020	57	157	41.53 ± 4.59	1439.75 ± 569.09	8,832,161	1.47 ± 0.75 ^a^	1.12 ± 0.77 ^a^	71.69 ± 11.17 ^a^	38.59 ± 7.72 ^bc^
2020−2021	58	153	42.81 ± 4.39	1481.23 ± 474.16	10,192,337	1.70 ± 1.35 ^a^	1.35 ± 1.21 ^a^	66.31 ± 19.72 ^a^	48.75 ± 27.27 ^c^

^1^ The 2018–2019 spawning season was characterized by high tides and turbid waters, resulting in juvenile *A. scapularis* morbidity in the fall and winter seasons. Therefore, biometry was not performed before the 2018 spawning season to avoid handling stress on broodstock, which could cause egg resorption or mortality, as observed in similar situations with yellow croaker (*Cilus gilberti*) broodstock at the same aquaculture center.

## Data Availability

All data are provided either within the article or in Supplementary Materials.

## Supplementary Materials

The following supporting information can be downloaded at https://www.mdpi.com/article/10.3390/ani15111579/s1.
